# The contribution of microscopy to targeting antimalarial treatment in a low transmission area of Tanzania

**DOI:** 10.1186/1475-2875-5-4

**Published:** 2006-01-20

**Authors:** Hugh Reyburn, John Ruanda, Ombeni Mwerinde, Chris Drakeley

**Affiliations:** 1 Dept. of Infectious and Tropical Disease, London School of Hygiene and Tropical Medicine, Keppel St, London WCIE 7HT, UK; 2 Joint Malaria Programme, PO Box 2228, KCMC, Moshi, Tanzania; 3 District Medical Officer, Njombe District, Tanzania; 4 Kilimanjaro Christian Medical Centre, Moshi, Tanzania

## Abstract

**Background:**

There is a need for improved targeting of antimalarial treatment if artemisinin combination therapy is to be successfully introduced in Africa. This study aimed to explore why malaria slides are requested and how their results guide treatment decisions in an area of low transmission of *P. falciparum*.

**Methods:**

Outpatients attending a district hospital in a highland area of Tanzania were studied over a 3-week period. Clinical and social data were collected from patients who had been prescribed an antimalarial or sent for a malaria slide. Hospital slides were re-read later by research methods.

**Results:**

Of 1,273 consultations 132(10%) were treated presumptively for malaria and 214(17%) were sent for a malaria slide; only 13(6%) of these were reported positive for *P. falciparum *but 96(48%) of the 201 slide-negative cases were treated for malaria anyway. In a logistic regression model, adults (OR 3.86, P < 0.01), a history of fever (OR1.72, P = 0.03) and a longer travel time to the clinic (OR 1.77 per hour travelled, P < 0.01) independently predicted the request for a malaria slide. Only a history of a cough predicted (negatively) the prescription of an antimalarial with a negative slide result (OR 0.44, P < 0.01). The sensitivity and specificity of hospital slide results were 50% and 96% respectively.

**Conclusion:**

Progress in targeting of antimalarials in low malaria transmission settings is likely to depend on consistent use of malaria microscopy and on the willingness of health workers to be guided by negative slide results. Further studies are needed to identify how this can be achieved.

## Background

In Tanzania, as in a number of African countries, existing antimalarial treatment (sulphadoxine-pyrimethamine, SP) is being replaced by artemesinin combination treatment (ACT) as first line treatment for non-severe malaria. However, at 5–10 times the cost of SP, its introduction creates an urgent need to review the diagnostic and treatment practices that have evolved over many years of cheap and safe antimalarials [1-3].

Where there is no access to microscopy presumptive diagnosis of malaria (fever with no obvious alternative cause) is widely practiced and results in a large degree of unnecessary use of antimalarials, especially at low transmission and in older children and adults in endemic areas [4]. Where available, microscopy offers a way forward but there is evidence that it has little impact as health workers frequently prescribe antimalarials in spite of a negative result [5]. Anecdotal reports also suggest that many patients are treated presumptively for malaria even when microscopy is available and it is not clear if slides are requested to confirm or exclude malaria or if patient demand or ability to pay for the slide also influence the decision to request a slide. There are few studies that have addressed these questions especially in low transmission settings in spite of the fact that approximately a third of the population of Africa lives in such areas [6]. This study was thus conducted in the outpatient clinic of a district hospital in a low transmission area of Tanzania to understand more about why a malaria slide is requested and how the results guide treatment.

## Methods

### The study area

Njombe town (population 42,332) is situated in the southern highlands of Tanzania at an altitude of between 1,800 and 1,920 metres and with a rainy season between November and May. Economic activity in the area consists of subsistence agriculture with some commercial farming of pyrethrum and coffee.

The 'Malaria Assessment of Risk in Africa' initiative (using projected rainfall and temperature) classifies the level of *P. falciparum *transmission in the Njombe area as 'unstable or absent' [7]. A survey of 100 consecutive children under the age of 15 years who were waiting for a medical consultation in Njombe Hospital in July 2004 found that only 4% were positive for *P. falciparum *[8].

Njombe District hospital serves the population of the town and surrounding villages, most of which are served by a dispensary without malaria microscopy. According to routinely collected data in 2003 there were 53,546 outpatient consultations, 19,812(37%) of which resulted in a diagnosis of malaria; 10,165 blood slides were examined in the hospital laboratory by 4 trained staff supported by 2 unqualified assistants. The mean number of slides/working day/slide reader was 10.6(SD 2.0). Malaria slides were charged at approximately $0.2 (U.S.) each for patients over the age of 5 years.

### Clinical and social data collection

All patients exiting from a medical out-patient consultation during a 3-week period and who had been prescribed an antimalarial or who had a request for a malaria slide were identified. Following consenting procedures, data were collected on age, sex, village of residence, distance and travel time to the clinic and the presence within the previous 2 days of cough, fever, vomiting or diarrhoea, 'body pain', or any other symptom. Possible indicators of socio-economic status were identified from local interviews and from opinions of researchers in Tanzania. Data on 8 items (household possession of a car, motorbike, bicycle, radio, mobile phone, television, fridge and any bed net) were analysed for correlation; household possession of a bicycle or bed net were dropped due to poor correlation and the remaining factors were found to highly correlate (alpha = 0.77) and added to produce a socio-economic score.

Patients were identified on exit from the review consultation and any changes to their medication were noted. Any patient who appeared too ill to participate was referred back to the clinic medical staff and not included in the study.

Data were collected by locally recruited research assistants who were not part of the normal hospital staff.

### Laboratory data collection

Blood slide results were tracked at the end of each day and the results were recorded from the laboratory results register, although it was not possible to determine if there had been an error in transcribing results to the register. Blood slides in the hospital laboratory were stained with Giemsa and 50 high power thick film fields were examined before declaring a slide to be negative, with no system of reporting parasite densities. All clinic blood slides were marked with the patient study number and retained for later research-quality reading where the number of *P. falciparum *asexual parasites per 200 leucocytes was counted on Giemsa-stained thick blood films. A slide was considered negative only after scanning 100 high power fields. All slides were read twice independently with a third reading if there was discordance, the majority result was accepted as final.

### Ethical approval and data management

Verbal consent of outpatient staff and written consent from patients was obtained in all cases. Ethical approval for the study was obtained from the review committees of Kilimanjaro Christian Medical Centre, Tanzania and the London School of Tropical Medicine and Hygiene.

Data were double-entered in Access (Microsoft Corporation, Redmond, WA) and statistical analysis was performed using STATA 8 (Stata Corporation, College Station, TX).

## Results

### Cases in the study, slide results and treatment given

During the 3-week period of the study there were there were 1,273 outpatient attendances, 240(19%) of which resulted in treatment for malaria. (Figure) Of the 346 patients who were recruited into the study approximately half (160, 46%) were under the age of 15 years. Females predominated in the 186 patients over the age of 15 years (129, 69%).

A blood slide was requested for 214 patients and all of these had a hospital laboratory slide result recorded. Thirteen (6%) of these were reported by the hospital laboratory as positive for *P. falciparum *asexual parasites and an additional 35(16%) were reported as positive for *P. falciparum *gametocytes. No other *Plasmodium *species was reported. Twelve (92%) of the cases reported as positive for asexual parasites were treated with an antimalarial. Thirty-three (94%) of the 35 patients with gametocytaemia reported by the hospital laboratory were also treated with an antimalarial and all were reported as negative for asexual parasites. Since gametocytes do not cause human illness these cases have been classified as 'slide negative'.

The sensitivity and specificity of hospital slide results for asexual parasitaemia (using the research slide result as the reference) were 50% (95% CI 43–57) and 96% (95% CI 93–98) respectively, and positive and negative predictive values were 31% (95% CI 26–37) and 98% (95% CI 96–100) respectively. Five slides were judged to be unreadable (all had been reported as negative by the hospital laboratory). *P. falciparum *asexual parasite densities of the 8 positive research slide results were 2 at <2000/μl, 2 at 2000–4,999/μl, and 4 at >5,000/μl. The research slide results identified only 3 positive slides for gametocytes, 2 of which were reported as negative by the hospital laboratory.

### Factors associated with blood slide requests and treatment decisions

In a logistic regression model the request for a malaria slide (as compared to presumptive treatment with no slide request) was independently predicted by being aged 15 years or over (OR 3.86, P < 0.01), reporting a history of fever in the previous 48 hours (OR 1.72, P = 0.03) and reporting a longer travel time to the clinic (a 77% increase in odds per hour travelled, P < 0.01). The request for a malaria slide request was not significantly associated with the socio-economic score (P = 0.26).(Table 2).

**Table 2:** Logistic regression model* of factors affecting the decision to request a malaria slide.

	**Antimalarial with no slide request (n = 132)**	**Slide request (n = 214)**	**Initial adjusted OR**	**P**	**Final adjusted OR**	**P**
Age 15 years or over, n (%)	47(36%)	139(65%)	4.03	<0.01	3.86	<0.01
Male (%)	58(44%)	78(37%)	1.01	0.16		
Distance from village/km^†^: median (IQR)^‡^	5(5–25) kms	10(5–25) kms	1.02	0.78		
Hours to travel to clinic: median (IQR)	1(1–2) hrs	2(1–2) hrs	1.95	<0.01	1.77	<0.01
Socio-economic score^§^: mean	0.97	1.05	1.16	0.26		
Number of days ill: median (IQR)	3(2–4) days	4(3–7) days	0.97	0.15		
Antimalarial used in previous 48 hrs: n (%)	3(2.3%)	8(3.8%)	0.90	0.89		
Fever in previous 48 hrs: n (%)	85(64%)	149(70%)	2.23	0.03	1.72	0.03
'Body pain' in previous 48 hrs: n (%)	92(70%)	168(79%)	1.06	0.84		
Cough in previous 48 hrs: n (%)	71(54%)	97(46%)	0.93	0.79		
Diarrhoea or vomiting in prev. 48 hrs: n (%)	55(43%)	82(38%)	0.87	0.60		
Other symptom in previous 48 hrs: n (%)	33(25%)	46(22%)	0.66	0.18		

*Logistic regression model with dependant variable = slide requested (1) or not requested (0), independent variables in model: aged 15 years or over, socio-economic score, distance to village, hours of travel to clinic, number of days ill, use of an antimalarial in 48 hrs before attendance, a report in the previous 48 hours of: fever, body pain, cough, diarrhoea or vomiting, or 'other' symptom (not specified). Variables were eliminated in a backwards fashion if p >= 0.1. All binary variables used the negative response as the baseline odds (i.e. OR = 1).

^†^Estimated distance between hospital outpatient clinic and village of residence.

^‡^IQR = Interquartile range

^§^Socio-economic score was derived from principal components analysis of reported possession in the household of the following: car, motorbike, radio, mobile phone, television, or refrigerator.

Of the 201 patients whose slide was reported as negative, 44(22%) were treated with an antimalarial only, 52(26%) with an antimalarial and an antibiotic, 68 (34%) with an antibiotic only, and 37(18%) with neither an antimalarial nor an antibiotic. Thus overall, 96(48%) of patients whose slide was reported as negative were treated with an antimalarial and of these 29(30%) did not report a fever within the previous 48 hours. Similarly, 47(36%) of those treated presumptively for malaria also denied having a fever within the previous 48 hours.

A logistic regression model using the same factors listed in Table 2 was used to test for an association with prescribing (compared to not prescribing) antimalarial treatment where the hospital slide result was reported as negative; the only factor found to be independently predictive was a negative association with a history of cough within the previous 48 hrs (OR 0.44, P < 0.01). Using the same variables to test the odds of being prescribed an antimalarial irrespective of whether a malaria slide was requested, only being under the age of 15 years was independently predictive (OR 2.25, p < 0.01).

## Discussion

In this study almost 20% of all outpatients were diagnosed and treated for malaria and routine data reported this proportion as almost 40% in the previous year, suggesting a possible moderating influence of the study itself. Blood slides were not collected from presumptively treated cases for fear of influencing clinical practice, but even if twice as many of these were positive compared to those who were sent for a malaria slide, well over 90% of treated cases did not have malaria, a finding consistent with other studies [4,9]. Hay et al. have estimated that over a third of the population of malaria-endemic countries in Africa lives under low or epidemic-prone transmission levels of malaria[6] and, although there are insufficient data to make a detailed extrapolation, it seems likely that a substantial proportion of avoidable antimalarial treatments in Africa is dispensed in these settings.

Health workers demonstrated unrealistic confidence in their ability to make a clinical diagnosis of malaria. Thus, as would be reasonable in a high transmission area, children tended to be treated presumptively. The failure to request a blood slide did not seem to be justified by excessive workload (although it is likely to be a factor in many settings) and data were not collected on the influence of waiting times on the request for a slide. However, if a blood slide is requested it is logical to expect the result to guide treatment decisions but almost half of those with a negative result were treated with an antimalarial anyway. If the clinical suspicion of malaria is strong then this might override the slide result (although logically one could ask why request a slide at all?) but 30% of slide-negative cases treated for malaria did not report a recent history of a fever. When requested, it was not clear if slide results were being used to confirm or exclude malaria; while children were almost 4 times less likely than adults to be slide-tested, those sent for a slide were more likely to have reported a recent history of fever than those treated presumptively.

Our results suggest that malaria slides in clinics may fulfil a social or ritual function. In this study, a slide request was more likely if patients had travelled further (suggesting patient motivation to attend a facility where microscopy was available) although there was no association with socio-economic status suggesting that the cost of the slide does not play a major role. Clearly, for health workers, malaria is a convenient and acceptable label for non-specific illness but little is known about the understanding of malaria that leads to such practice and whether the phenomenon is driven more by patients or health workers.

Presumptive diagnosis of malaria is an effective strategy to increase coverage of antimalarials in high transmission areas where malaria is common, the risk of progression to severe malaria is significant, and diagnostic facilities are lacking. In low transmission areas the justification for presumptive diagnosis is much less clear. Current Malaria Guidelines in Tanzania[10] do not vary by transmission intensity (although the most recent version does distinguish between those over and under the age of 5 years) but the introduction of ACTs creates a strong economic case that in low transmission areas treatment for malaria should, wherever possible, be restricted to parasitologically proven cases [1]. Such an approach will have additional benefits in that patients are more likely to be treated for the actual cause of their illness and generation of more reliable routine data can support improved health planning as well as give early warning of epidemics. How to achieve a situation where health workers respect negative slide results is unclear; in a teaching hospital it has been possible in patients over the age of 5 years[11] but, as far as we are aware, there are no similar reports in less supervised settings where the majority of patients are seen. While clear national guidelines, improved laboratory standards and RDTs all have a role, it is optimistic to expect these to have a major impact without addressing prescribing behaviour and patient expectations about which there is currently little in-depth knowledge.

The accuracy of slide reading in this study was low although the negative predictive value (being dependent on prevalence) was still in excess of 95%, providing a high likelihood that a decision to withhold an antimalarial on the basis of a negative slide would be correct. In spite of this there is clearly an urgent need to improve laboratory standards; Bates et al.[12] have shown that this can be achieved with a relatively simple system of quality control and further improvements are likely if laboratory staff more frequently see that their results actually influence clinical decisions.

## Conclusion

Reducing the overuse of antimalarials is an important and complex challenge. The criteria that are used to request a malaria slide are inconsistent and, where requested, negative results are often disregarded. If RDTs are introduced on a wide scale in Africa similar questions regarding consistent use, quality control and use of the results will need to be addressed.

## Authors' contributions

HR and CD conceived of the study and its design and drafted the manuscript. JR coordinated the data collection and OM analysed the data; both JR and OM made critical comments to the manuscript.

**Figure 1:**
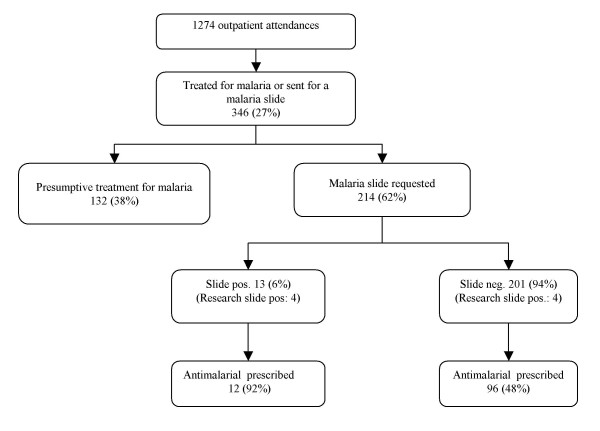
Cases in the study by malaria slide result and treatment given.

**Table 1:** Total outpatient attendances during the 21-day period of the study according to primary diagnosis.

**Diagnosis**	*Age <5 yr	**Age 5+ yr**	**Total**
Acute respiratory infection	184(40%)	174(21%)	358(28%)
Malaria	96(21%)	144(18%)	240(19%)
Diarrhoea	58(13%)	59(7%)	117(9%)
Skin infection	23(5%)	21(3%)	44(3%)
Intestinal helminths	17(4%)	9(1%)	26(2%)
Ear infection	7(2%)	7(1%)	14(1%)
Urinary tract infection	3(1%)	11(1%)	14(1%)
Anaemia	2(0.4%)	10(1%)	12(1%)
Other	70(15%)	378(46%)	448(35%)

**Total**	460	813	1,273

*Age groups determined by routine reporting of outpatient attendances according to Ministry of Health guidelines.
